# The Combination of IFN β and TNF Induces an Antiviral and Immunoregulatory Program via Non-Canonical Pathways Involving STAT2 and IRF9

**DOI:** 10.3390/cells8080919

**Published:** 2019-08-17

**Authors:** Mélissa K. Mariani, Pouria Dasmeh, Audray Fortin, Elise Caron, Mario Kalamujic, Alexander N. Harrison, Diana I. Hotea, Dacquin M. Kasumba, Sandra L. Cervantes-Ortiz, Espérance Mukawera, Adrian W. R. Serohijos, Nathalie Grandvaux

**Affiliations:** 1 CRCHUM—Centre Hospitalier de l’Université de Montréal, Montréal, QC H2X 0A9, Canada; 2 Department of Biochemistry and Molecular Medicine, Faculty of Medicine, Université de Montréal, Montréal, QC H3T 1J4, Canada; 3 Centre Robert Cedergren en Bioinformatique et Génomique, Université de Montréal, Montréal, QC H3T 1J4, Canada; 4 Department of Microbiology and Immunology, McGill University, Montréal, QC H3A 2B4, Canada; 5 Department of Microbiology, Infectiology and Immunology, Faculty of Medicine, Université de Montréal, Montréal, QC H3T 1J4, Canada

**Keywords:** interferon, tumor necrosis factor, STAT, interferon regulatory factor, antiviral, autoimmunity, inflammation

## Abstract

Interferon (IFN) β and Tumor Necrosis Factor (TNF) are key players in immunity against viruses. Compelling evidence has shown that the antiviral and inflammatory transcriptional response induced by IFNβ is reprogrammed by crosstalk with TNF. IFNβ mainly induces interferon-stimulated genes by the Janus kinase (JAK)/signal transducer and activator of transcription (STAT) pathway involving the canonical ISGF3 transcriptional complex, composed of STAT1, STAT2, and IRF9. The signaling pathways engaged downstream of the combination of IFNβ and TNF remain elusive, but previous observations suggested the existence of a response independent of STAT1. Here, using genome-wide transcriptional analysis by RNASeq, we observed a broad antiviral and immunoregulatory response initiated in the absence of STAT1 upon IFNβ and TNF costimulation. Additional stratification of this transcriptional response revealed that STAT2 and IRF9 mediate the expression of a wide spectrum of genes. While a subset of genes was regulated by the concerted action of STAT2 and IRF9, other gene sets were independently regulated by STAT2 or IRF9. Collectively, our data supports a model in which STAT2 and IRF9 act through non-canonical parallel pathways to regulate distinct pool of antiviral and immunoregulatory genes in conditions with elevated levels of both IFNβ and TNF.

## 1. Introduction

Interferon (IFN) β plays a critical role in the first line of defense against viruses, through its ability to induce a broad antiviral transcriptional response in virtually all cell types [1]. IFNβ also possesses key immunoregulatory functions that determine the outcome of the adaptive immune response against pathogens [1,2]. Over the years, in vitro and in vivo studies aimed at characterizing the mechanisms and the functional outcomes of IFNβ signaling were mostly performed in relation to single cytokine stimulation. This unlikely reflects physiological settings, as a plethora of cytokines are secreted in a specific situation. As a consequence, a cell rather simultaneously responds to a cocktail of cytokines to foster the appropriate transcriptional program. Response to IFNβ is no exception and is very context-dependent, particularly regarding the potential crosstalk with other cytokines. Elevated levels of IFNβ and Tumor Necrosis Factor (TNF) are found during the host response to viruses. Aberrant increased levels of both cytokines is also associated with a number of autoinflammatory and autoimmune diseases such as Systemic Lupus Erythematosus (SLE), psoriasis, and Sjögren’s syndrome [3]. While the cross-regulation of IFNβ and TNF is well documented [4,5,6], the functional crosstalk between these two cytokines remains poorly known.

IFNβ typically acts through binding to the IFNAR receptor (IFNAR1 and IFNAR2) leading to the Janus kinase (JAK)/signal transducer and activator of transcription (STAT) pathway involving JAK1- and Tyk2-mediated phosphorylation of STAT1 and STAT2, and to a lesser extent other STAT members in a cell-specific manner [7,8]. Phosphorylated STAT1 and STAT2, together with IFN Regulatory Factor (IRF) 9, form the IFN-stimulated gene factor 3 (ISGF3) complex that binds to the consensus IFN-stimulated response element (ISRE) sequences in the promoter of hundreds of IFN stimulated genes (ISGs) [9]. Formation of the ISGF3 complex is considered a hallmark of the engagement of the type I IFN response and, consequently, the requirement of STAT1 in a specific setting has become a marker of the engagement of type I IFN signaling [7,10]. However, in recent years, this paradigm has started to be challenged with accumulating evidence demonstrating the existence of non-canonical JAK-STAT signaling that mediates type I IFN responses [8,11].

Synergism between IFNβ and TNF was shown to enhance the antiviral response to Vesicular Stomatitis Virus (VSV), Myxoma virus, and paramyxovirus infections [12,13,14]. Gene expression analyses showed that IFNβ and TNF synergistically regulate hundreds of genes induced by individual cytokines alone, but also drive a specific delayed transcriptional program composed of genes that are either not responsive to IFNβ or TNF separately or are only responsive to either one of the cytokine [4,13]. The signaling mechanisms engaged downstream of the costimulation with IFNβ and TNF remained elusive, but it is implicitly assumed that the fate of the gene expression response requires that both IFNβ- and TNF-induced signaling pathways exhibit significant crosstalk. Analysis of the enrichment of specific transcription factors binding sites in the promoters of a panel of genes synergistically induced by IFNβ and TNF failed to give a clue about the specificity of the transcriptional regulation of these genes [13]. We previously showed that the *DUOX2* gene belongs to the category of delayed genes that are remarkably induced to high levels in response to the combination of IFNβ and TNF in lung epithelial cells [8]. We found that *DUOX2* expression required STAT2 and IRF9 but not STAT1, suggesting that STAT2 and IRF9 activities might segregate in an alternative STAT1-independent pathway that could be involved in gene regulation downstream of IFNβ and TNF [14].

In the present study, we aimed to fully characterize the transcriptional profile of the delayed response to IFNβ and TNF that occurs independently of STAT1 and evaluate the role of STAT2 and IRF9 in the regulation of this response. We found that the costimulation by IFNβ and TNF induces a broad set of antiviral and immunoregulatory genes in the absence of STAT1. We also report the differential regulation of distinct subsets of IFNβ− and TNF-induced genes by STAT2 and IRF9. While IFNβ and TNF act in part through the concerted action of STAT2 and IRF9, specific sets of genes were only regulated by either STAT2 or IRF9. Altogether, our findings uncovered non-canonical STAT2 and/or IRF9-dependent pathways that coexist to regulate distinct pools of antiviral and immunoregulatory genes in a context of IFNβ and TNF crosstalk.

## 2. Materials and Methods

### 2.1. Cell Culture and Stimulation

A549 cells (American Type Culture Collection, ATCC) were grown in F-12 nutrient mixture (Ham) medium supplemented with 10% heat-inactivated fetal bovine serum (HI-FBS) and 1% L-glutamine. The 2ftGH fibrosarcoma cell line and the derived STAT1-deficient U3A cell line, a generous gift from Dr. G. Stark, Cleveland, USA [15], were grown in DMEM medium supplemented with 10% HI-FBS or HI-Fetal Clone III (HI-FCl) and 1% L-glutamine. U3A cells stably expressing STAT1 were generated by transfection of the STAT1 alpha flag pRc/CMV plasmid (Addgene plasmid #8691; a generous gift from Dr. J. Darnell, Rockfeller University, USA [16,17]) and selection with 800 μg/ml Geneticin (G418). Monoclonal populations of U3A stably expressing STAT1 cells were isolated. A pool of two clones, referred to as U3A-STAT1, was used in the experiments to mitigate the clonal effects. U3A-STAT1 cells were maintained in culture in DMEM supplemented with 10% HI-FCl, 1% Glu, and 200 μg/mL G418. All cell lines were cultured without antibiotics except for the selection of stable cells. All media and supplements were from Gibco (Life Technologies, Grand Island, NY, USA), with the exception of HI-FCl, which was from HyClone (Logan, UT, USA). Mycoplasma contamination was excluded by regular analysis using the MycoAlert Mycoplasma Detection Kit (Lonza, Basel, Switzerland). Cells were stimulated with IFNβ (1000 U/mL, PBL Assay Science, Piscataway, NJ, USA), TNF (10 ng/mL, R&D Systems, Minneapolis, MN, USA) or IFNβ (1000 U/mL) +TNF (10 ng/mL) for the indicated times.

### 2.2. siRNA Transfection

The sequences of non-targeting control (Ctrl) and STAT2- and IRF9-directed RNAi oligonucleotides (Dharmacon, Lafayette, CO, USA) have previously been described in [14]. U3A cells at 30% confluency were transfected using the Oligofectamine transfection reagent (Life Technologies-Thermofisher, Carlsbad, CA, USA). RNAi transfection was pursued for 48 h before stimulation.

### 2.3. Immunoblot Analysis

Cells were lysed on ice using Nonidet P-40 lysis buffer as fully detailed in [18]. Whole-cell extracts (WCE) were quantified using the Bradford protein assay (Bio-Rad, Hercules, CA, USA), resolved by SDS-PAGE and transferred to nitrocellulose membrane before analysis by immunoblot. Membranes were incubated with the following primary antibodies: anti-actin Cat #MAB1501 from Millipore (Burlington, MA, USA), anti-IRF9 Cat #610285 from BD Transduction Laboratories (San Jose, CA, USA), and anti-STAT1-P-Tyr701 Cat #9171, anti-STAT2-P-Tyr690 Cat #4441, anti-STAT1 Cat #9172, anti-STAT2 Cat #4594, all from Cell Signaling (Danvers, MA, USA), before further incubation with horseradish peroxidase (HRP)-conjugated secondary antibodies (KPL, Gaithersburg, MD, USA or Jackson Immunoresearch Laboratories, West Grove, PA, USA). Antibodies were diluted in PBS containing 0.5% Tween and either 5% nonfat dry milk or BSA. Immunoreactive bands were visualized by enhanced chemiluminescence (Western Lightning Chemiluminescence Reagent Plus, Perkin-Elmer Life Sciences Waltham, MA, USA) using a LAS4000mini CCD camera apparatus (GE Healthcare, Mississauga, ON, Canada).

### 2.4. RNA Isolation and qRT-PCR Analyses

Total RNA was prepared using the RNAqueous-96 Isolation Kit (Invitrogen-Thermo Fisher, Carlsbad, CA, USA) following the manufacturer’s instructions. Total RNA (1 µg) was subjected to reverse transcription using the QuantiTect Reverse Transcription Kit (Qiagen, Toronto, ON, Canada). Quantitative PCR were performed using either Fast start SYBR Green Kit (Roche, Indianapolis, IN, USA) for *MX1*, *IDO*, *APOBEC3G*, *CXCL10*, *NOD2*, *PKR*, *IRF1, IFIT1* and *IL8* or TaqMan Gene Expression Assays (Life Technologies-Thermo Fisher) for *DUOX2*, *IFI27*, *SERPINB2*, *IL33*, *CCL20*, *ISG20*. Sequences of oligonucleotides and probes used in PCR reactions are described in Supplemental Table S4. Data collection was performed on a Rotor-Gene 3000 Real Time Thermal Cycler (Corbett Research, Mortlake, Australia). Gene inductions were normalized over S9 levels, measured using Fast start SYBR Green Kit or TaqMan probe as necessary. Fold induction of genes was determined using the ΔΔCt method [19]. All qRT-PCR data are presented as the mean ± standard error of the mean (SEM).

### 2.5. RNA-Sequencing (RNASeq)

Total RNA prepared as described above was quantified using a NanoDrop Spectrophotometer ND-1000 (NanoDrop Technologies, Inc., Wilmington, DE, USA) and its integrity was assessed using a 2100 Bioanalyzer (Agilent Technologies, Santa Clara, CA, USA). Libraries were generated from 250 ng of total RNA using the NEBNext poly(A) magnetic isolation module and the KAPA stranded RNA-Seq library preparation kit (Kapa Biosystems, Wilmington, MA, USA), as per the manufacturer’s recommendations. TruSeq adapters and PCR primers were purchased from IDT. Libraries were quantified using the Quant-iT™ PicoGreen^®^ dsDNA Assay Kit (Molecular Probes, Eugene, OR, USA) and the Kapa Illumina GA with Revised Primers-SYBR Fast Universal kit (Kapa Biosystems). Average size fragment was determined using a LabChip GX (Perkin-Elmer Life Sciences, Waltham, MA, USA) instrument. Massively parallel sequencing was carried out on an Illumina HiSeq 2500 sequencer (Illumina Inc., San Diego, CA, USA). Read counts were obtained using HTSeq. Reads were trimmed from the 3’ end to have a Phred score of at least 30. Illumina sequencing adapters were removed from the reads and all reads were required to have a length of at least 32bp. Trimming and clipping was performed using Trimmomatic [20]. The filtered reads were aligned to the Homo-sapiens assembly GRCh37 reference genome. Each read set was aligned using STAR [21] and merged using Picard (http://broadinstitute.github.io/picard/). For all samples, the sequencing resulted in more than 29 million clean reads (ranging from 29 to 44 million reads) after removing low quality reads and adaptors. The reads were mapped to the total of 63,679 gene biotypes including 22,810 protein-coding genes. The non-specific filter for 1 count-per million reads (CPM) in at least three samples was applied to the reads and 14,254 genes passed this criterion.

### 2.6. Bioinformatics Analysis

Differential transcripts analysis. A reference-based transcript assembly was performed, which allows the detection of known and novel transcripts isoforms, using Cufflinks [22], merged using Cuffmerge (cufflinks/AllSamples/merged.gtf) and used as a reference to estimate transcript abundance and perform differential analysis using Cuffdiff and Cuffnorm tool to generate a normalized data set that includes all the samples. The fragments per kilobase million (FPKM) values calculated by Cufflinks were used as input. The transcript quantification engine of Cufflinks, Cuffdiff, was used to calculate transcript expression levels in more than one condition and test them for significant differences. To identify a transcript as being differentially expressed, Cuffdiff tests the observed log-fold-change in its expression against the null hypothesis of no change (i.e., the true log-fold-change is zero). Because of measurement errors, technical variability, and cross-replicate biological variability might result in an observed log-fold-change that is non-zero, Cuffdiff assesses significance using a model of variability in the log-fold-change under the null hypothesis. This model is described in detail in [23]. The differential gene expression analysis was performed using DESeq [24] and edgeR [25] within the R Bioconductor packages. Genes were considered differentially expressed between two group if they met the following requirement: fold change (FC) > ±1.5, *p* < 0.05, false discovery rate (FDR) < 0.05.

Enrichment of gene ontology (GO). GO enrichment analysis amongst differentially expressed genes (DEGs) was performed using Goseq [26] against the background of full human genome (hg19). GO-terms with adjusted *p* value < 0.05 were considered significantly enriched.

Clustering of DEGs. We categorized the DEGs according to their response upon silencing of siSTAT2 and siIRF9; categories are listed as A to I (Figure 2E). Then to determine relationship between these categories, we calculated the distance of centers of different categories. For each gene, we transformed siSTAT2 and siIRF9 FC to deviation from the mean FC of the category the respective gene belongs to using the equation: *FC_new_* = *FC_old_* − *ε* (*FC_category_*). The parameter ε was estimated to give the perfect match between predefined categories (A to I) and clustering based on Euclidean distance. Results were plotted as a heatmap.

Modular transcription analysis. The *tmod* package in R [27] was used for modular transcription analysis. In brief, each transcriptional module is a set of genes that shows coherent expression across many biological samples [28,29]. Modular transcription analysis then calculates significant enrichment of a set of foreground genes, here DEGs, in pre-defined transcriptional module compared to a reference set. For transcriptional modules, we used a combined list of 606 distinct functional modules encompassing 12,712 genes, defined by Chaussabel et al. [30] and Li et al. [31], as the reference set in *tmod* package (Supplemental Table S5). The hypergeometric test devised in *tmodHGtest* was used to calculate enrichments and p-values employing Benjamini-Hochberg correction [32] for multiple sampling. All the statistical analyses and graphical presentations were performed in R [33].

### 2.7. Virus Titration by Plaque Assay

Quantification of VSV infectious virions was achieved through methylcellulose plaque forming unit assays. U3A and U3A-STAT1 cells were either left untreated or stimulated with IFNβ or IFNβ + TNF for 30 h. Cells were then infected with Vesicular Stomatitis Virus (VSV)-GFP (kindly provided by Dr. J. Bell, University of Ottawa, Canada) at a multiplicity of infection (MOI) of 5 for 1 h in serum-free medium (SFM). Cells were then washed twice with SFM and further cultured in DMEM medium containing 2% HI-FCl. The supernatants were harvested at 12 h post-infection and serial dilutions were used to infect confluent Vero cells (ATCC) for 1 h in SFM. The medium was then replaced with 1% methylcellulose in DMEM containing 10% HI-FCl. Two days post-infection, GFP-positive plaques were detected using a Typhoon Trio apparatus and quantified using the ImagequantTL software (GE Healthcare, Mississauga, ON, Canada).

### 2.8. Luciferase Gene Reporter Assay

U3A or U3A-STAT1 cells at 90% confluency were cotransfected with 100 ng of one of the following CXCL10 promoter containing firefly luciferase reporter plasmids (generously donated by Dr. David Proud, Calgary, [34]), CXCL10prom-972pb-pGL4 (full length −875/+97 promoter), CXCL10prom-376pb-pGL4 (truncated −279/+97 promoter), CXCL10prom972pb-ΔISRE(3)-pGL4 (full length promoter with ISRE(3) site mutated), together with 50 ng of pRL-null renilla-luciferase expressing plasmid (internal control). Transfection was performed using Lipofectamine 2000 (Life Technologies-Thermo Fisher) using a 1:2 DNA to lipofectamine ratio. At 8 h post-transfection, cells were stimulated for 16 h with either IFNβ or IFNβ + TNF. Firefly and renilla luciferase activities were quantified using the Dual-luciferase reporter assay system (Promega Corporation, Madison, WI, USA). Luciferase activities were calculated as the luciferase/renilla ratio and were expressed as fold over the non-stimulated condition.

### 2.9. Statistical Analyses

Statistical analyses of qRT-PCR and luciferase assay results were performed using the Prism v7 or v8 software (GraphPad Software, San Diego, CA, USA) using the tests indicated in the figure legends. Statistical significance was evaluated using the following *p* values: *p* < 0.05 (*), *p* < 0.01 (**), *p* < 0.001 (***) or *p* < 0.0001 (****). Differences with a *p*-value < 0.05 were considered significant. Statistical analysis of the RNASeq data is described in the Bioinformatics analysis section above.

### 2.10. Data and Software Availability

The entire set of RNAseq data has been submitted to the Gene Expression Omnibus (GEO) database (http://www.ncbi.nlm.nih.gov/geo) under accession number GEO: GSE111195.

## 3. Results

### 3.1. Distinct Induction Profiles of Antiviral and Immunoregulatory Genes in Response to IFNβ, TNF and IFNβ + TNF

**Figure 1:**
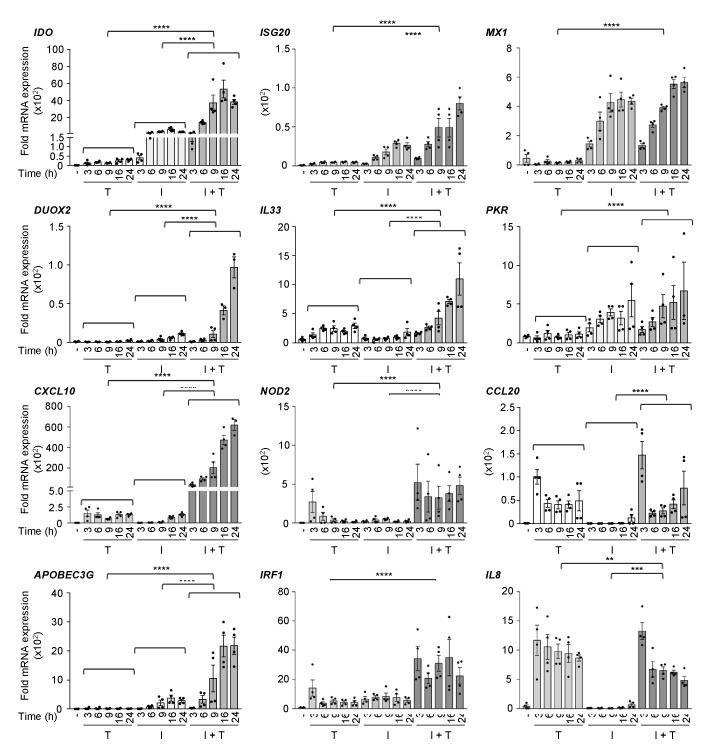
Expression of a panel of antiviral and immunoregulatory genes in response to IFNβ, TNF, and IFNβ + TNF. A549 cells were stimulated with either TNF (T), IFNβ (I), or costimulated with IFNβ + TNF (I + T) for the indicated times. Quantification of mRNA was performed by qRT-PCR and expressed as fold expression after normalization to the S9 mRNA levels using the ΔΔCt method. Mean +/− SEM, *n* ≥ 3. Statistical comparison of TNF vs. IFNβ + TNF and IFNβ vs. IFNβ + TNF was conducted using two-way ANOVA with Tukey’s post-test. *p* < 0.01 (**), *p* < 0.001 (***) or *p* < 0.0001 (****).

First, to validate previous observations, we sought to determine the induction profile by the combination of IFNβ and TNF of a selected panel of immunoregulatory and antiviral genes that had previously been shown to be regulated by IFNβ or TNF alone. A549 cells, in which we previously documented the synergistic action of IFNβ and TNF on the *DUOX2* gene, were stimulated either with IFNβ, TNF, or IFNβ + TNF for various times between 3–24 h and the relative mRNA expression levels were quantified by qRT-PCR. Analysis of the expression of the selected genes revealed distinct profiles of response to IFNβ, TNF, or IFNβ + TNF (Figure 1). *IDO*, *DUOX2*, *CXCL10*, *APOBEC3G, ISG20,* and *IL33* exhibited synergistic induction in response to IFNβ + TNF compared to IFNβ or TNF alone. Expression in response to IFNβ + TNF increased over time, with maximum expression levels observed between 16 and 24 h. While *NOD2* and *IRF1* induction following stimulation with IFNβ + TNF was also significantly higher than upon IFNβ or TNF single cytokine stimulation, they exhibited a steady-state induction profile starting as early as 3 h. *MX1* and *PKR*, two typical IFNβ-inducible ISGs, were found induced by IFNβ + TNF similarly to IFNβ alone. *CCL20* responded to IFNβ + TNF with a kinetic and amplitude similar to TNF, but was not responsive to IFNβ alone. *IL8* expression was not induced by IFNβ, but was increased by TNF starting at 3 h and remained steady until 24 h. In contrast to other genes, *IL8* induction in response to IFNβ + TNF was significantly decreased compared to TNF alone. Overall, these results confirm previous reports that a subset of antiviral and immunoregulatory genes is greatly increased in response to IFNβ + TNF compared to either IFNβ or TNF alone.

### 3.2. Workflow for Genome-Wide Characterization of the Delayed Transcriptional Program Induced by IFNβ+TNF in the Absence of STAT1

**Figure 2:**
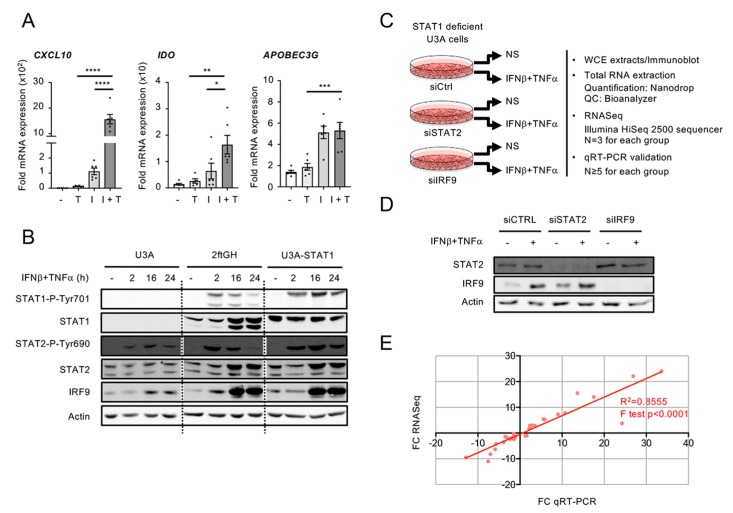
Experimental design used to study the STAT1-independent delayed transcriptional program induced by the combination of IFNβ and TNF. (**A**) 2ftGH cells were stimulated with either TNF (T), IFNβ (I), or costimulated with IFNβ + TNF (I + T) for 24 h. Quantification of mRNA was performed by qRT-PCR and expressed as fold expression after normalization to the S9 mRNA levels using the ΔΔCt method. Mean +/− SEM, *n* ≥ 5. Statistical comparison was conducted using one-way ANOVA with Tukey’s post-test. *p* < 0.05 (*), *p* < 0.01 (**), *p* < 0.001 (***), or *p* < 0.0001 (****). (**B**) U3A (STAT1-deficient), 2ftGH (parental STAT1-positive) cells and U3A-STAT1 cells (U3A cells stably reconstituted with STAT1) were left untreated or stimulated with IFNβ + TNF for the indicated times. WCE (whole cell extracts) were analyzed by SDS-PAGE followed by immunoblot using anti STAT1-P-Tyr701, total STAT1, STAT2-P-Tyr690, total STAT2, IRF9, or actin antibodies. (**C**–**E**) U3A cells were transfected with siCTRL, siSTAT2, or siIRF9 before being left untreated (NS) or stimulated with IFNβ + TNF for 24 h. (**C**) The schematic describes the workflow of sample preparation and analysis. (**D**) WCE were analyzed by SDS-PAGE followed by immunoblot using anti STAT2, IRF9, and actin antibodies. (**E**) Graph showing the correlation between fold-changes (FC) measured by RNASeq and qRT-PCR for 13 randomly selected genes. Data from siCTRL NS vs. siCTRL IFNβ +TNF, siSTAT2 IFNβ +TNF vs. siCTRL IFNβ + TNF, siIRF9 IFNβ + TNF vs. siCTRL IFNβ + TNF conditions were used.

In a previous study, we provided evidence supporting the existence of a STAT1-independent, but STAT2- and IRF9-dependent, pathway engaged downstream of IFNβ + TNF [14]. Here, using the STAT1-deficient human U3A cell line [15], we aimed to fully characterize the STAT1-independent transcriptional program induced by IFNβ + TNF. The human U3A cell line was derived by mutagenesis from the 2ftGH cells [15], in which a synergistic response to IFNβ + TNF can be observed on a subset of genes (Figure 2A). Two hallmarks of STAT2 and IRF9 activation, i.e., STAT2 Tyr690 phosphorylation and induction of IRF9, were observed in the U3A cells following stimulation with IFNβ + TNF, although to reduced levels compared to the parental 2ftGH cells expressing endogenous STAT1 (Figure 2B). This observation implies that the activation of STAT2 and IRF9 depends to a large extent on the STAT1-dependent canonical pathway, but that a significant response occurs in the absence of STAT1. Therefore, the human U3A cell model is suitable for specifically studying STAT1-independent, but STAT2- and IRF9-dependent, gene expression in response to IFNβ + TNF.

To profile the genome wide transcriptional program induced by the combination of IFNβ and TNF in the absence of STAT1 and define the role of STAT2 and IRF9, the U3A cells were transfected with Control (Ctrl)−, STAT2- or IRF9-RNAi and further left untreated or stimulated with IFNβ (1000 U/mL) + TNF (10 ng/mL) for 24 h (Figure 2C). Efficient silencing was confirmed by immunoblot (Figure 2D). Total RNA was isolated and analyzed by RNA sequencing (n = 3 for each group detailed in Figure 2B) on an Illumina HiSeq2500 platform. To validate the expression profile of genes in RNASeq results, the fold changes (FC) of 13 genes randomly selected were analyzed by qRT-PCR in each experimental groups, i.e., siCTRL non-stimulated (NS) vs. siCTRL IFNβ + TNF, siCTRL IFNβ + TNF vs. siSTAT2 IFNβ + TNF and siCTRL IFNβ + TNF vs. siIRF9 IFNβ + TNF. A positive linear relationship between RNASeq and qRT-PCR FC was observed (Figure 2E).

### 3.3. A Broad Antiviral and Immunoregulatory Transcriptional Signature is Induced by IFNβ + TNF in the Absence of STAT1

**Figure 3:**
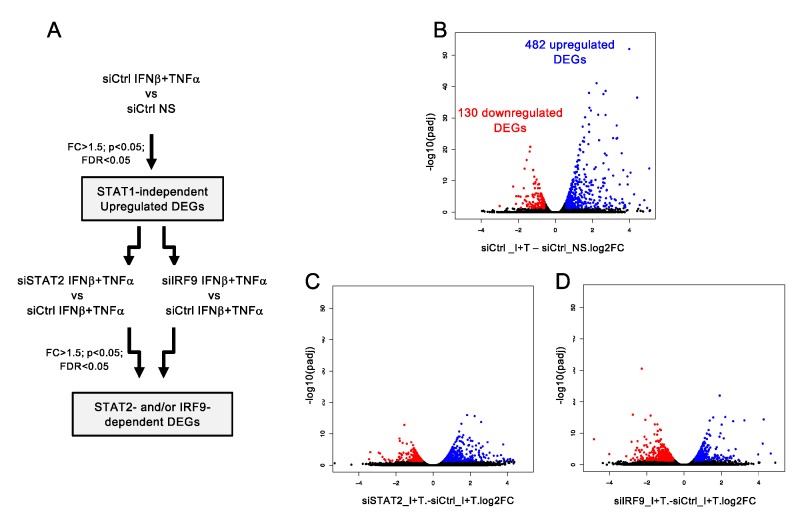
Analysis of STAT1-independent IFNβ + TNF-induced DEGs. (**A**) Diagram describing the bioinformatics analysis strategy used to determine STAT1-independent differentially expressed genes (DEGs) and their regulation by STAT2 and IRF9. (**B**) Volcano plots of the fold-change (FC) vs. adjusted *p*-value of IFNβ + TNF (Ι + Τ) vs. non-stimulated (NS) siCtrl conditions. (**C**) Volcano plots of the fold-change vs. adjusted *p*-value of siSTAT2 IFNβ + TNF vs. siCTRL IFNβ + TNF (I + T) conditions. (**D**) Volcano plots of the fold-change vs. adjusted *p*-value of siIRF9 IFNβ + TNF vs. siCTRL IFNβ + TNF conditions.

To identify differentially expressed genes (DEGs) upon IFNβ + TNF stimulation in the absence of STAT1, comparison between non-stimulated (NS) and IFNβ + TNF-stimulated control cells was performed (Figure 3A). In total, 612 transcripts, including protein-coding transcripts, pseudogenes and long non-coding RNA (lncRNA), were significantly different (FC > 1.5, *p* < 0.05, FDR < 0.05) in IFNβ + TNF vs. NS. Among these, 482 DEGs were upregulated and 130 were downregulated (Figure 3B; See Supplemental Table S1 for a complete list of DEGs).

**Figure 4:**
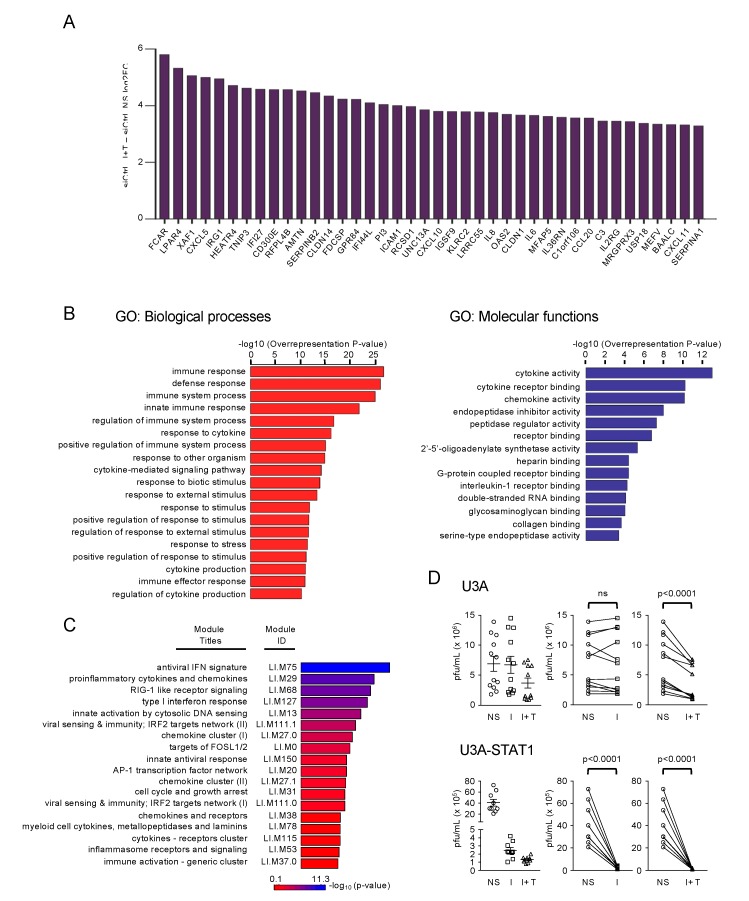
Functional characterization of STAT1-independent IFNβ + TNF-induced DEGs. (**A**) Top forty IFNβ + TNF- upregulated DEGs. (**B**) Gene ontology (GO) enrichment analysis of the differentially upregulated genes in IFNβ + TNF vs. non-stimulated siCtrl conditions based on the Biological Processes and Molecular Function categories. Top enriched terms are shown and the full list is available in Supplemental Table S2. (**C**) Modular transcription analysis of upregulated DEGs. Eighteen enriched modules are shown. The full list of enriched modules is available in Supplemental Table S3. (**D**) U3A and STAT1-rescued U3A-STAT1 cells were stimulated with IFNβ (I) or IFNβ + TNF (I + T) for 30 h before infection with VSV at a MOI of 5 for 12 h. The release of infectious viral particles was quantified by plaque forming unit (pfu) assay. The left graphs show dot-plots of all stimulations. Statistical comparisons were performed on the “before and after” plots (displayed on the right of dot-plot graphs) using ratio paired *t*-tests.

To identify the Biological Processes (BP) and Molecular Functions (MF) induced by IFNβ + TNF independently of STAT1, we further analyzed the upregulated DEGs. The top 40 upregulated DEGs are shown in Figure 4A. We subjected the complete list of upregulated DEGs (Supplemental Table S1) through Gene Ontology (GO) enrichment analysis. The top enriched GO BP (*p* < 10^−10^) and MF, are depicted in Figure 4B (See Supplemental Table S2 for a complete list of enriched GO). The majority of the top enriched BPs were related to cytokine production and function (response to cytokine, cytokine-mediated signaling pathway, cytokine production, and regulation of cytokine production), immunoregulation (immune response, immune system process, innate immune response, and regulation of immune system process) and host defense response (defense response, response to other organism, 2’-5’-oligoadenylate synthetase activity and dsRNA binding). Fourteen MF categories were found enriched in IFNβ + TNF. The top enriched MFs were related to cytokine and chemokine functions (cytokine activity, cytokine receptor binding, chemokine activity, and Interleukin 1-receptor binding). Other enriched MF included peptidase related functions (endopeptidase inhibitor activity, peptidase regulator activity, and serine-type endopeptidase activity).

To gain deeper insight into the relevance of the STAT1-independent IFNβ + TNF-induced transcripts towards immunological and host defense responses, we conducted a modular transcription analysis of upregulated DEGs against 606 immune-related functional modules. These modules were previously defined from co-clustered gene sets built via an unbiased data-driven approach as detailed in the material and methods section [30,31]. STAT1-independent IFNβ + TNF-induced DEGs showed significant enrichment in 37 modules (See Supplemental Table S3 for a complete list of enriched modules). Six of these modules were associated with virus sensing/Interferon antiviral response, including LI.M75 (antiviral IFN signature), LI.M68 (RIG-I-like receptor signaling), LI.M127 (type I interferon response), LI.M111.0 and LI.M111.1 (IRF2 target network), and LI.M150 (innate antiviral response) (Figure 4C). Additionally, six modules were associated with immunoregulatory functions, including LI.M29 (proinflammatory cytokines and chemokines), LI.M27.0 and LI.M27.1 (chemokine cluster I and II), LI.M38 (chemokines and receptors), LI.M115 (cytokines receptors cluster), and LI.M37.0 (immune activation - generic cluster) (Figure 4C). Module analysis also showed enriched AP-1 transcription factor-related network modules, LI.M20 and LI.M0, and cell cycle and growth arrest LI.M31 module.

GO enrichment and modular transcription analyses were suggestive of an antiviral response being induced by IFNβ + TNF. To demonstrate the physiological relevance of STAT1-independent gene expression, we evaluated the capacity of IFNβ + TNF to restrict virus replication in the absence of STAT1. Previous study has shown that infection by Vesicular Stomatitis Virus (VSV) is sensitive to IFNβ, but not to TNF, in 2ftGH-derived cells [35]. Thus, U3A cells were stimulated with IFNβ alone or IFNβ + TNF and further infected with (VSV). While VSV replicated similarly in untreated U3A cells and in cells treated with IFNβ, treatment with IFNβ + TNF significantly limited VSV replication (Figure 4D). As a control of the efficiency of IFNβ alone treatment, we observed a significant antiviral response when STAT1 expression was stably restored to endogenous levels in U3A cells (U3A-STAT1) (Figure 2B and Figure 4D). Collectively, these data demonstrate that the combination of IFNβ + TNF induces an efficient antiviral and immunoregulatory transcriptional response in the absence of STAT1.

### 3.4. Clustering of STAT1-Independent IFNβ + TNF Induced DEGs According to their Regulation by STAT2 and IRF9

**Figure 5:**
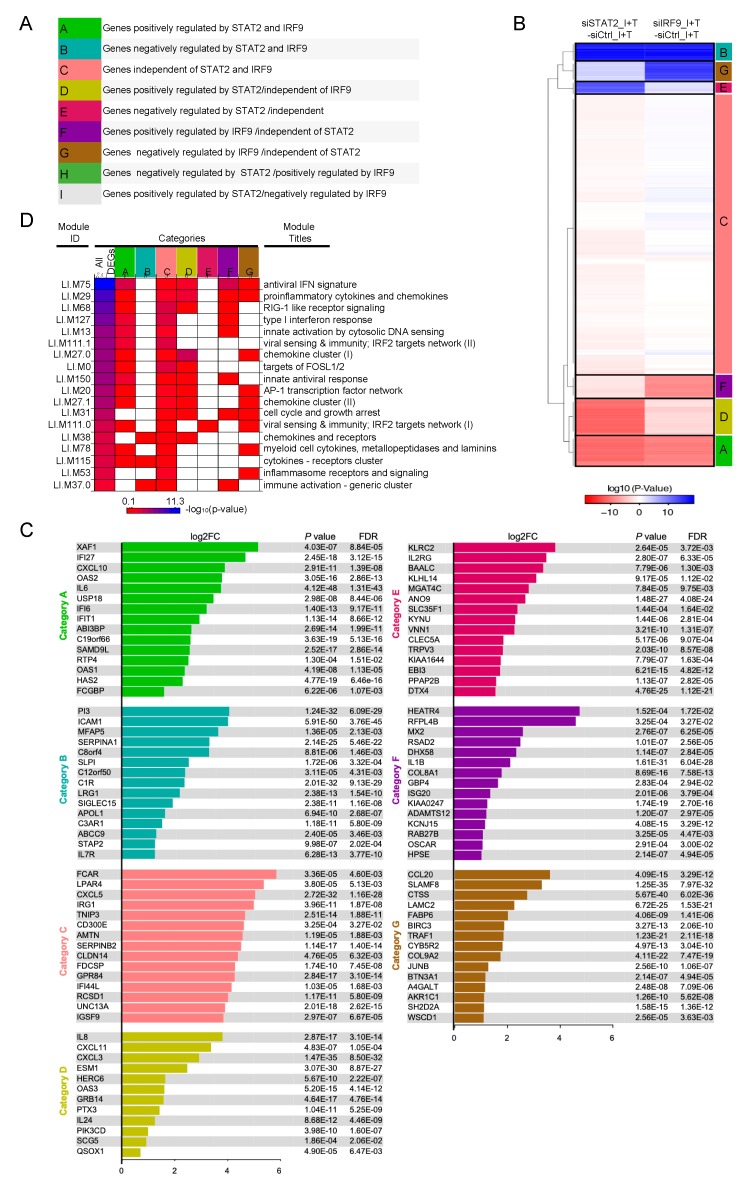
Clustering of IFNβ + TNF-induced DEGs according to their regulation by STAT2 and IRF9. (**A**) Theoretical categories in which IFNβ + TNF-induced DEGs can be segregated based on their potential individual regulation by STAT2 and IRF9. (**B**) Hierarchical clustering of the categories of DEG responses according to their regulation by STAT2 and IRF9. Euclidean distance metric is used for the construction of distance matrix and the categories are used as a priori input into clustering algorithm as detailed in Materials and Methods. (**C**) Top 15 induced DEGs (Log2FC, siCTRL NS vs. siCTRL IFNβ + TNF) in each category identified in (**B**) are displayed. Note that category D contains only twelve genes, so all genes are shown. The full list of genes is available in Supplemental Table S1. (**D**) Diagram showing enriched transcription modules in each gene category.

Having shown that IFNβ + TNF induce a broad antiviral and immunoregulatory transcriptional response independently of STAT1, we next sought to gain insight into the role of STAT2 and IRF9. To do so, we compared transcripts levels in siSTAT2_IFNβ + TNF vs. siCTRL_IFNβ + TNF and siIRF9_IFNβ + TNF vs. siCTRL_IFNβ + TNF conditions (Figure 3A and Supplemental Table S1). Volcano plots revealed that a fraction of IFNβ + TNF-induced DEGs were significantly (FC > 1.5, *p* < 0.05, FDR < 0.05) downregulated or upregulated upon silencing of STAT2 (Figure 3C) or IRF9 (Figure 3D). Nine distinct theoretical categories of DEGs can be defined based on their potential individual behavior across siSTAT2 and siIRF9 groups (Categories A–I, Figure 5A): a gene can either be downregulated upon STAT2 and IRF9 silencing, indicative of a positive regulation by STAT2 and IRF9 (Category A); conversely, a gene negatively regulated by STAT2 and IRF9 would exhibit upregulation upon STAT2 and IRF9 silencing (Category B); genes that do not exhibit significant differential expression in siSTAT2 and siIRF9 groups would be classified as STAT2 and IRF9 independent (Category C); IRF9-independent genes could exhibit positive (Category D) or negative (Category E) regulation by STAT2; conversely, STAT2-independent genes might be positively (Category F) or negatively (Category G) regulated by IRF9; lastly, STAT2 and IRF9 could have opposite effects on a specific gene regulation (Category H and I). Based on a priori clustering of RNASeq data we found that in the absence of STAT1 IFNβ + TNF-induced DEGs clustered into only seven of the nine possible categories (Figure 5B). The top 15 upregulated DEGs of each category are shown in Figure 5C. The complete list of genes in each category is available in Supplemental Table S1. Amongst the 482 DEGs, 163 genes exhibited either inhibition or upregulation following silencing of STAT2 and/or IRF9 (Categories B–G). A large majority of upregulated DEGs, i.e., 319 out of the 482 DEGs, were not significantly affected by either STAT2 or IRF9 silencing, and were therefore classified as STAT2/IRF9-independent (Figure 5B). No genes were found in Category H and only one gene was found in Category I.

To functionally interpret these clusters, we applied the modular transcription analysis to each of the categories to assess for the specific enrichment of the functional modules found associated with IFNβ + TNF-upregulated DEGs (Figure 5D). First, most modules, except LI.M31 (cell cycle and growth arrest), LI.M38 (chemokines and receptors), LI.M37.0 (immune activation - generic cluster), and LI.M53 (inflammasome receptors and signaling), were enriched in the category of DEGs positively regulated by STAT2 and IRF9 (Category A). Conversely, the cluster negatively regulated by STAT2 and IRF9 (Category B) exclusively contains enriched LI.M38 (chemokines and receptors), LI.M37.0 (immune activation - generic cluster) and LI.M115 (cytokines receptors cluster). The cluster of IRF9-independent genes that are negatively regulated by STAT2 (Category E) only exhibited enrichment in the virus sensing/IRF2 target network LI.M111.0 module, while the IRF9-independent/STAT2-positively regulated cluster (Category D) encompasses antiviral and immunoregulatory functions. The STAT2-independent but IRF9 positively regulated transcripts (Category F) were mainly enriched in modules associated with the IFN antiviral response, including LI.M75 (antiviral IFN signature), LI.M68 (RIG-I-like receptor signaling), LI.M127 (type I interferon response), and LI.M150 (innate antiviral response). Finally, the STAT2-independent but IRF9 negatively regulated cluster (Category G) was mostly enriched in modules associated with immunoregulatory functions, including LI.M29 (proinflammatory cytokines and chemokines), LI.M27.0 and LI.M27.1 (chemokine cluster I and II), LI.M78 (myeloid cell cytokines), but also with cell cycle and growth arrest (LI.M31) and inflammasome receptors and signaling (LI.M53). Of note, all modules were found enriched in the cluster of genes induced independently of STAT2 and IRF9 (Category C), pointing to a broad function of this pathway(s) in the regulation of the antiviral and immunoregulatory program elicited by IFNβ + TNF. Altogether, these observations reveal that STAT2 and IRF9 are involved in the regulation of a subset of the genes induced in response to the co-stimulation by IFNβ and TNF in the absence of STAT1. Importantly, our results also reveal that STAT2 and IRF9 act in part in a concerted manner, but also independently in distinct non-canonical pathways, to regulate specific subsets of the IFNβ + TNF-induced DEGs.

### 3.5. Differential Regulation of CXCL10 in Response to IFNβ and IFNβ+TNF

**Figure 6:**
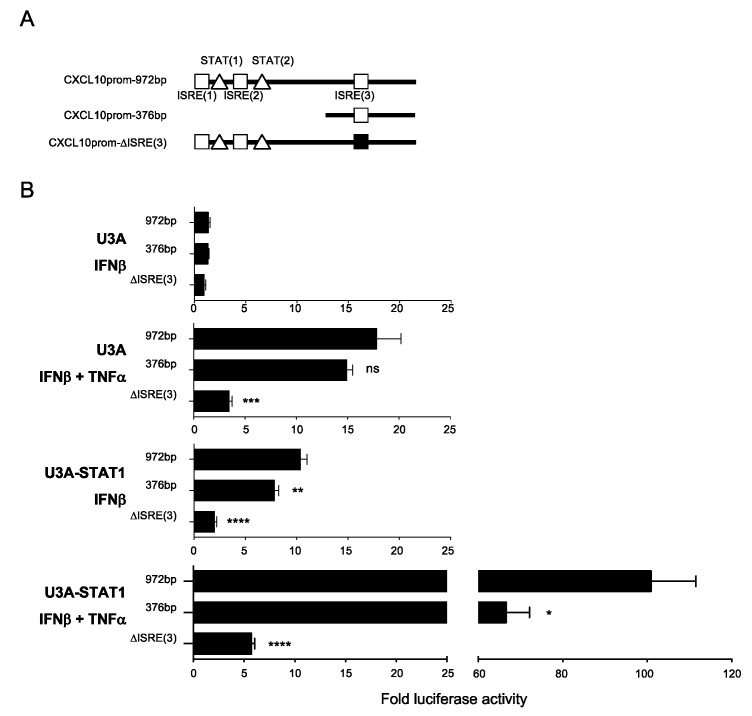
Analysis of the *CXCL10* promoter regulation in response to IFNβ + TNF vs. IFNβ. (**A**) Schematic representation of the *CXCL10* promoter (CXCL10prom) luciferase constructs used in this study indicating the main transcription factors consensus binding sites. (**B**) U3A and U3A-STAT1 cells were transfected with the indicated CXCL10prom-luciferase reporter constructs and either left untreated or stimulated with IFNβ or IFNβ + TNF. Relative luciferase activities were measured at 16 h post-stimulation and expressed as fold over the corresponding unstimulated condition. Mean +/− SEM, n = 6. Statistical analyses were performed using an unpaired t-test comparing each promoter to the CXCL10prom-972bp construct. *p* < 0.01 (**), *p* < 0.001 (***), and *p* < 0.0001 (****).

The canonical ISGF3 complex mediates ISGs transcriptional regulation through binding to ISRE consensus sequences [7]. Identification of DEGs upregulated by IFNβ+TNF in a STAT1-independent, but STAT2 and IRF9-dependent, manner raised the question of the ISRE site usage compared to the ISGF3 pathway. To address this question, we studied the regulation of the *CXCL10* gene promoter that was found induced by STAT2 and IRF9 in the absence of STAT1 in response to IFNβ and TNF (Supplemental Table S1 and Figure 5C), but is also inducible by IFNβ alone in the presence of STAT1 (Figure 1 and Figure 2A). The *CXCL10* promoter contains three ISRE sites. We used full-length wild-type (972bp containing the three ISRE sites), truncated (376bp containing only the ISRE (3) site) or mutated (972bp containing a mutated ISRE (3) site) *CXCL10* promoter luciferase (*CXCL10*prom-Luc) reporter constructs (Figure 6A) to determine the ISRE consensus site(s) requirement. U3A and STAT1-rescued U3A-STAT1 cells were transfected with the *CXCL10*prom-Luc constructs and further stimulated with IFNβ or IFNβ+TNF to monitor the canonical ISGF3-dependent and the STAT1-independent responses. In STAT1-deficient U3A cells, IFNβ was unable to activate the *CXCL10*prom reflecting the dependence on the ISFG3 pathway. In contrast, induction of *CXCL10*prom was significantly induced when cells were stimulated with IFNβ +TNF. This induction in the absence of STAT1 was not altered by the deletion of ISRE (1) and (2) sites, but was significantly impaired when the ISRE (3) site was mutated (Figure 6B). STAT1 expression rescue led to a higher induction of *CXCL10*prom by IFNβ + TNF. Additionally, IFNβ-mediated induction of *CXCL10*prom was restored, albeit to a much lower extent than IFNβ +TNF. The activation of the *CXCL10*prom in the presence of STAT1 involved both the distal ISRE (1) and/or ISRE (2) sites and the proximal ISRE (3) site (Figure 6B). Altogether, this shows that the STAT1-independent, but STAT2/IRF9-dependent, pathway mediates gene expression through a restricted ISRE site usage compared to the ISGF3-dependent regulation.

## 4. Discussion

Previous studies have described that IFNβ and TNF synergize to elicit a specific delayed transcriptional program that differs from the one induced by either cytokine alone [13,36]. The mechanisms underlying the transcriptional induction of genes specifically regulated by IFNβ and TNF remain poorly defined. The present study was specifically designed to document the functional relevance of a previously observed delayed gene expression induced by IFNβ in the presence of TNF in the absence of STAT1 [14] and to document the role of STAT2 and IRF9 in this response.

The observation that STAT2 and IRF9 activation in response to IFNβ + TNF is reduced in STAT1-deficient U3A cells compared to the wild-type 2ftGH parental cells and that IFNβ + TNF-mediated induction of the STAT2- and IRF9-dependent *CXCL10* promoter exhibits partial dependence on STAT1 support a model in which a canonical ISGF3 pathway is engaged downstream of the costimulation. Importantly, it also implied the existence of a STAT2- and/or IRF9-dependent transcriptional response occurring in the absence of STAT1. The human STAT1-deficient U3A cell model offered a unique opportunity to specifically pinpoint this STAT1-independent response. In this model, genome wide RNA sequencing highlighted that the transcriptional program induced by IFNβ + TNF in the absence of STAT1 encompasses a wide range of immunoregulatory and antiviral functions. The functional relevance of this response was confirmed by the observation that the treatment with IFNβ + TNF induced an antiviral state capable of restricting VSV replication in the absence of STAT1. This points to a significant role of the STAT1-independent pathway in the establishment of the antiviral state induced by the synergistic action of IFNβ and TNF that enhances the restriction of VSV (Figure 4D and [12]), Myxoma virus [13], and paramyxoviruses [14]. Although previous reports have shown that type I IFNs, mostly IFNα, alone can trigger STAT1-independent responses [8], we neither observed establishment of an IFNβ-induced antiviral state against VSV, nor activation of the *CXCL10* promoter in the absence of STAT1 in our model (Figure 4D and Figure 6).

We previously reported that IFNβ + TNF induces the *DUOX2* gene via a STAT2- and IRF9-dependent pathway in the absence of STAT1 [14]. To what extent this pathway contributes to the STAT1-independent transcriptional response elicited by IFNβ + TNF remained to be addressed. Here, we demonstrate that IFNβ + TNFα-induced DEGs segregate into seven categories that reflect distinct contributions of STAT2 and/or IRF9, thereby highlighting an unexpected heterogeneity of the STAT1-independent pathways engaged downstream of IFNβ + TNF. Importantly, only one anecdotic gene was found in categories implying inverse regulation by STAT2 and IRF9 (categories H and I) pointing to convergent functions of STAT2 and IRF9 when both are engaged in gene regulation. We can rule out that these distinct regulation mechanisms reflect specific induction profiles by IFNβ + TNF as *CXCL10*, *IL33*, *CCL20,* and *ISG20* all exhibit synergistic induction by IFNβ + TNF, but are differentially regulated by STAT2 and/or IRF9; while *CXCL10* is dependent on STAT2 and IRF9, *IL33* is independent on STAT2 and IRF9, and *CCL20* and *ISG20* are STAT2-independent but IRF9-dependent (Figure 5; Supplemental Table S1). Consistent with our previous observation [14], we found several STAT1-independent genes positively regulated by STAT2 and IRF9 (Category A). DEGs in this category encompass most of the functions induced in response to IFNβ + TNF, with the notable exception of cell cycle and growth arrest and inflammasome and receptor signaling functions. Genes negatively regulated by STAT2 and IRF9 were also identified (Category B). Formation of an alternative STAT2/IRF9-containing complex mediating gene expression in the absence of STAT1 [37,38,39,40,41] has been reported, but with limited DNA-binding affinity for the typical ISRE sequence [37]. The existence of a STAT2/IRF9 complex is also supported by our recent observation of a high affinity of IRF9 for STAT2 with an equilibrium dissociation constant (Kd) of 10 nM [42]. A recent report of experiments, performed in murine bone marrow-derived macrophages proposes a model in which murine STAT2/IRF9 complex drives basal expression of ISGs, while IFNβ-inducible expression of ISGs depends on a switch to the ISGF3 complex [43]. This differs from our results as silencing of either STAT2 or IRF9 did not alter basal gene expression (Supplemental Figure S1). Further analysis of the *CXCL10* promoter demonstrates a restricted usage of ISRE sites by the STAT2/IRF9 pathway compared to the ISGF3 pathway. Further large-scale studies will be needed to identify the parameters allowing binding of ISGF3, but not STAT2/IRF9, to specific ISRE sequences upon IFNβ + TNF.

The observation that some IFNβ + TNF-induced genes were solely dependent on STAT2 (either positively or negatively) but not on IRF9, (Categories D and E) is a rare genome wide demonstration of gene regulation by STAT2 independently of STAT1 and IRF9. Previous reports have identified ISGF3-independent, STAT2-dependent genes but the association with IRF9 was not formally excluded [44,45,46,47]. STAT2 was shown to associate with STAT3 and STAT6, but it is not clear whether IRF9 is also part of these alternative complexes [44,46]. Transcriptional module analyses demonstrated that the functional distribution of genes negatively regulated by STAT2 is very limited compared to other categories; only a virus-sensing module was enriched in this category. In contrary, IRF9-independent genes positively regulated by STAT2 mediate broader antiviral and immunoregulatory functions.

ISGF3-independent functions of IRF9 have been proposed based on the study of IRF9 deficiencies [11,48]. However, IRF9 target genes in these contexts have been barely documented. Intriguingly, Li et al. [49] studied IFNα-induced genes and their dependency on the ISGF3 subunits. While they confirmed previous studies showing that IFNα can trigger a delayed and sustained ISG response via an ISGF3-independent pathway, it is very striking that they did not find STAT1- and STAT2-independent but IRF9-dependent genes. All identified IRF9-dependent genes were either STAT2- or STAT1-dependent. This result greatly differs with our study. Here, we found several IFNβ + TNF-induced DEGs independent of STAT1 and STAT2, but positively or negatively regulated by IRF9 (Categories F and G). Typically, IRF9 is considered a positive regulator of gene transcription. However, our findings are consistent with recent reports documenting the role of IRF9 in the negative regulation of the TRIF/NF-κB transcriptional response [50] or the expression of SIRT1 in acute myeloid leukemia cells [51]. The molecular mechanisms underlying gene regulation by IRF9 without association with either STAT1 or STAT2 remain to be elucidated. To the best of our knowledge, no alternative IRF9-containing complex has yet been described.

Our analysis showed that a large number of genes were induced by IFNβ + TNF independently of STAT2 and IRF9 (Category C). All transcriptional modules were enriched in this category pointing to a major role of this pathway in the establishment of a host defense and immunoregulatory response. The STAT2 and IRF9 independent genes does not solely reflects induction by TNF alone. For instance, *APOBEC3G* that is amongst the STAT2- and IRF9- independent genes is not induced by TNF alone (Figure 2A). While NF-κB, a downstream effector of the TNF receptor, is an obvious candidate for the regulation of these DEGs, this might fall short in explaining the synergistic action of IFNβ + TNF as we did not observe enhanced NF-κB activation compared to TNF alone [14]. Alternatively, the potential role of AP-1 is supported by the finding that the AP-1 transcription network module is enriched amongst IFNβ + TNF-induced DEGs. However, this module is not restricted to genes regulated independently of STAT2 and IRF9. It is also worth noting that two modules of IRF2-target genes were enriched, although again not specifically in the STAT2- and IRF9-independent category. A similar crosstalk was reported between IFNα and TNF in macrophages resulting in increased colocalized recruitment of IRF1 and p65 to the promoter of a subset of genes [52]. However, while IRF1 was found synergistically induced by IFNβ + TNF at early stages (Figure 1), we did not observe significant induction of IRF1 in the absence of STAT1 by RNASeq (Supplemental Table S1) or qRT-PCR (data not shown), suggesting that IRF1 is unlikely to be involved in our system. Further studies will be required to uncover these STAT2- and IRF9-independent pathways.

**Figure 7:**
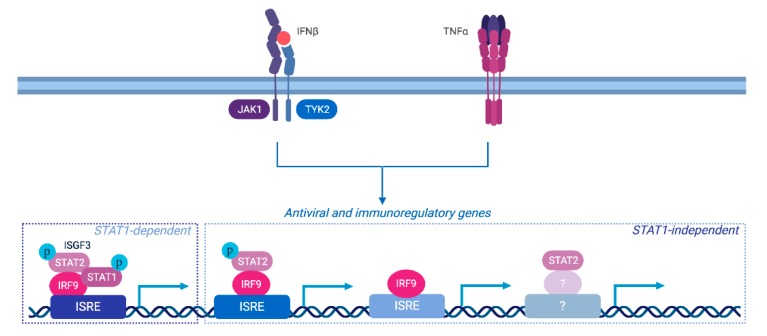
Role of distinct STAT2 and/or IRF9-dependent pathways in the regulation of distinct subset of antiviral and immunoregulatory genes in response to IFNβ and TNF. Our data supports a model in which multiple pathways participate to the synergistic action of IFNβ + TNF. While the STAT1-dependent pathway, likely ISFG3, is engaged downstream of IFNβ and TNF, STAT1-independent pathways are also involved in the control of the delayed gene expression. STAT2 and IRF9 act not only in a concerted fashion, likely as a complex, but also independently. IRF9 is known to act as the DNA-binding subunit of the ISGF3 complex and therefore likely mediates binding of STAT2/IRF9 complexes and of alternative complexes devoid of STAT2. The mechanisms of STAT2-dependent regulation of gene expression remains to be characterized.

This study provides novel insight into the molecular pathways leading to delayed antiviral and immunoregulatory gene expression in conditions where elevated levels of both IFNβ and TNF are present. Altogether our results demonstrate that in addition to the engagement of an ISGF3-dependent canonical response, a broad transcriptional program is elicited independently of STAT1, and support a model in which STAT2 and IRF9 contribute to the regulation of this response through non-canonical parallel pathways involving their concerted or independent action (Figure 7).

Consistent with accumulating evidence [8], these distinct STAT2 and IRF9 actions most likely result from the formation of specific complexes that coexist with ISGF3 upon IFNβ and TNF stimulation. Studies are underway to biochemically solve the complexity of the dynamic and specific mechanisms of activation of the alternative STAT2 and/or IRF9-containing complexes in a wild-type cell context to further characterize the transcriptional response induced by IFNβ and TNF.

## Supplementary Materials

The following are available online at https://www.mdpi.com/2073-4409/8/8/919/s1, Table S1: List of I+T_ DEG; Table S2: List of GO_I+T _DEG; Table S3: Module analysis_DEG; Table S4: MatetMeth_qPCR; Table S5: list_all_modules; Figure S1: qRT-PCR analysis of STAT2 and IRF9 regulated DEGs.

## References

[1] McNab F., Mayer-Barber K., Sher A., Wack A., O’Garra A. (2015). Type I interferons in infectious disease. Nat. Rev. Immunol..

[2] Tomasello E., Pollet E., Vu Manh T.P., Uze G., Dalod M. (2014). Harnessing Mechanistic Knowledge on Beneficial Versus Deleterious IFN-I Effects to Design Innovative Immunotherapies Targeting Cytokine Activity to Specific Cell Types. Front. Immunol..

[3] Lee-Kirsch M.A. (2017). The Type I Interferonopathies. Annu. Rev. Med..

[4] Yarilina A., Park-Min K.H., Antoniv T., Hu X., Ivashkiv L.B. (2008). TNF activates an IRF1-dependent autocrine loop leading to sustained expression of chemokines and STAT1-dependent type I interferon-response genes. Nat. Immunol..

[5] Palucka A.K., Blanck J.P., Bennett L., Pascual V., Banchereau J. (2005). Cross-regulation of TNF and IFN-alpha in autoimmune diseases. Proc. Natl. Acad. Sci. USA.

[6] Tliba O., Tliba S., Da Huang C., Hoffman R.K., DeLong P., Panettieri R.A., Amrani Y. (2003). Tumor necrosis factor alpha modulates airway smooth muscle function via the autocrine action of interferon beta. J. Biol. Chem..

[7] Au-Yeung N., Mandhana R., Horvath C.M. (2013). Transcriptional regulation by STAT1 and STAT2 in the interferon JAK-STAT pathway. JAKSTAT.

[8] Fink K., Grandvaux N. (2013). STAT2 and IRF9: Beyond ISGF3. JAKSTAT.

[9] Schneider W.M., Chevillotte M.D., Rice C.M. (2014). Interferon-stimulated genes: A complex web of host defenses. Annu. Rev. Immunol..

[10] Levy D.E., Marie I.J., Durbin J.E. (2011). Induction and function of type I and III interferon in response to viral infection. Curr. Opin. Virol..

[11] Majoros A., Platanitis E., Kernbauer-Holzl E., Rosebrock F., Muller M., Decker T. (2017). Canonical and Non-Canonical Aspects of JAK-STAT Signaling: Lessons from Interferons for Cytokine Responses. Front. Immunol..

[12] Mestan J., Brockhaus M., Kirchner H., Jacobsen H. (1988). Antiviral activity of tumour necrosis factor. Synergism with interferons and induction of oligo-2′, 5′-adenylate synthetase. J. Gen. Virol..

[13] Bartee E., Mohamed M.R., Lopez M.C., Baker H.V., McFadden G. (2009). The addition of tumor necrosis factor plus beta interferon induces a novel synergistic antiviral state against poxviruses in primary human fibroblasts. J. Virol..

[14] Fink K., Martin L., Mukawera E., Chartier S., De Deken X., Brochiero E., Miot F., Grandvaux N. (2013). IFNbeta/TNFalpha synergism induces a non-canonical STAT2/IRF9-dependent pathway triggering a novel DUOX2 NADPH oxidase-mediated airway antiviral response. Cell Res..

[15] McKendry R., John J., Flavell D., Muller M., Kerr I.M., Stark G.R. (1991). High-frequency mutagenesis of human cells and characterization of a mutant unresponsive to both alpha and gamma interferons. Proc. Natl. Acad. Sci. USA.

[16] Horvath C.M., Wen Z., Darnell J.E. (1995). A STAT protein domain that determines DNA sequence recognition suggests a novel DNA-binding domain. Genes Dev..

[17] Schindler C., Fu X.Y., Improta T., Aebersold R., Darnell J.E. (1992). Proteins of transcription factor ISGF-3: One gene encodes the 91-and 84-kDa ISGF-3 proteins that are activated by interferon alpha. Proc. Natl. Acad. Sci. USA.

[18] Robitaille A.C., Mariani M.K., Fortin A., Grandvaux N. (2016). A High Resolution Method to Monitor Phosphorylation-dependent Activation of IRF3. J. Vis. Exp..

[19] Dussault A.A., Pouliot M. (2006). Rapid and simple comparison of messenger RNA levels using real-time PCR. Biol. Proced. Online.

[20] Bolger A.M., Lohse M., Usadel B. (2014). Trimmomatic: A flexible trimmer for Illumina sequence data. Bioinformatics.

[21] Dobin A., Davis C.A., Schlesinger F., Drenkow J., Zaleski C., Jha S., Batut P., Chaisson M., Gingeras T.R. (2013). STAR: Ultrafast universal RNA-seq aligner. Bioinformatics.

[22] Roberts A., Pimentel H., Trapnell C., Pachter L. (2011). Identification of novel transcripts in annotated genomes using RNA-Seq. Bioinformatics.

[23] Trapnell C., Hendrickson D.G., Sauvageau M., Goff L., Rinn J.L., Pachter L. (2013). Differential analysis of gene regulation at transcript resolution with RNA-seq. Nat. Biotechnol..

[24] Anders S., Huber W. (2010). Differential expression analysis for sequence count data. Genome Biol..

[25] Robinson M.D., McCarthy D.J., Smyth G.K. (2010). edgeR: A Bioconductor package for differential expression analysis of digital gene expression data. Bioinformatics.

[26] Young M.D., Wakefield M.J., Smyth G.K., Oshlack A. (2010). Gene ontology analysis for RNA-seq: Accounting for selection bias. Genome Biol..

[27] Weiner J., Domaszewska T. (2016). tmod: An R package for general and multivariate enrichment analysis. PeerJ Prepr..

[28] Bar-Joseph Z., Gerber G.K., Lee T.I., Rinaldi N.J., Yoo J.Y., Robert F., Gordon D.B., Fraenkel E., Jaakkola T.S., Young R.A. (2003). Computational discovery of gene modules and regulatory networks. Nat. Biotechnol..

[29] Chaussabel D., Baldwin N. (2014). Democratizing systems immunology with modular transcriptional repertoire analyses. Nat. Rev. Immunol..

[30] Chaussabel D., Quinn C., Shen J., Patel P., Glaser C., Baldwin N., Stichweh D., Blankenship D., Li L., Munagala I. (2008). A modular analysis framework for blood genomics studies: Application to systemic lupus erythematosus. Immunity.

[31] Li S., Rouphael N., Duraisingham S., Romero-Steiner S., Presnell S., Davis C., Schmidt D.S., Johnson S.E., Milton A., Rajam G. (2014). Molecular signatures of antibody responses derived from a systems biology study of five human vaccines. Nat. Immunol..

[32] Benjamini Y., Hochberg Y. (1995). Controlling the False Discovery Rate: A Practical and Powerful Approach to Multiple Testing. J. R. Stat. Soc. Ser. B (Methodol.).

[33] Ross I., Gentleman R. (1996). R: A language for data analysis and graphics. J. Comput. Graph. Stat..

[34] Zaheer R.S., Koetzler R., Holden N.S., Wiehler S., Proud D. (2009). Selective transcriptional down-regulation of human rhinovirus-induced production of CXCL10 from airway epithelial cells via the MEK1 pathway. J. Immunol..

[35] Davis A.M., Hagan K.A., Matthews L.A., Bajwa G., Gill M.A., Gale M., Farrar J.D. (2008). Blockade of virus infection by human CD4+ T cells via a cytokine relay network. J. Immunol..

[36] Bartee E., McFadden G. (2009). Human cancer cells have specifically lost the ability to induce the synergistic state caused by tumor necrosis factor plus interferon-beta. Cytokine.

[37] Bluyssen H.A., Levy D.E. (1997). Stat2 is a transcriptional activator that requires sequence-specific contacts provided by stat1 and p48 for stable interaction with DNA. J. Biol. Chem..

[38] Martinez-Moczygemba M., Gutch M.J., French D.L., Reich N.C. (1997). Distinct STAT structure promotes interaction of STAT2 with the p48 subunit of the interferon-alpha-stimulated transcription factor ISGF3. J. Biol. Chem..

[39] Gupta S., Jiang M., Pernis A.B. (1999). IFN-alpha activates Stat6 and leads to the formation of Stat2:Stat6 complexes in B cells. J. Immunol..

[40] Lou Y.J., Pan X.R., Jia P.M., Li D., Xiao S., Zhang Z.L., Chen S.J., Chen Z., Tong J.H. (2009). IRF-9/STAT2 [corrected] functional interaction drives retinoic acid-induced gene G expression independently of STAT1. Cancer Res..

[41] Abdul-Sater A.A., Majoros A., Plumlee C.R., Perry S., Gu A.D., Lee C., Shresta S., Decker T., Schindler C. (2015). Different STAT Transcription Complexes Drive Early and Delayed Responses to Type I IFNs. J. Immunol..

[42] Rengachari S., Groiss S., Devos J., Caron E., Grandvaux N., Panne D. (2018). Structural basis of STAT2 recognition by IRF9 reveals molecular insights into ISGF3 function. Proc. Natl. Acad. Sci. USA.

[43] Platanitis E., Demiroz D., Schneller A., Fischer K., Capelle C., Hartl M., Gossenreiter T., Muller M., Novatchkova M., Decker T. (2019). A molecular switch from STAT2-IRF9 to ISGF3 underlies interferon-induced gene transcription. Nat. Commun..

[44] Ghislain J.J., Fish E.N. (1996). Application of genomic DNA affinity chromatography identifies multiple interferon-alpha-regulated Stat2 complexes. J. Biol. Chem..

[45] Brierley M.M., Marchington K.L., Jurisica I., Fish E.N. (2006). Identification of GAS-dependent interferon-sensitive target genes whose transcription is STAT2-dependent but ISGF3-independent. FEBS J..

[46] Wan L., Lin C.W., Lin Y.J., Sheu J.J., Chen B.H., Liao C.C., Tsai Y., Lin W.Y., Lai C.H., Tsai F.J. (2008). Type I IFN induced IL1-Ra expression in hepatocytes is mediated by activating STAT6 through the formation of STAT2: STAT6 heterodimer. J. Cell Mol. Med..

[47] Perry S.T., Buck M.D., Lada S.M., Schindler C., Shresta S. (2011). STAT2 mediates innate immunity to Dengue virus in the absence of STAT1 via the type I interferon receptor. PLoS Pathog..

[48] Suprunenko T., Hofer M.J. (2016). The emerging role of interferon regulatory factor 9 in the antiviral host response and beyond. Cytokine Growth Factor Rev..

[49] Li W., Hofer M.J., Songkhunawej P., Jung S.R., Hancock D., Denyer G., Campbell I.L. (2017). Type I interferon-regulated gene expression and signaling in murine mixed glial cells lacking signal transducers and activators of transcription 1 or 2 or interferon regulatory factor 9. J. Biol. Chem..

[50] Zhao X., Chu Q., Cui J., Huo R., Xu T. (2017). IRF9 as a negative regulator involved in TRIF-mediated NF-kappaB pathway in a teleost fish, Miichthys miiuy. Mol. Immunol..

[51] Tian W.L., Guo R., Wang F., Jiang Z.X., Tang P., Huang Y.M., Sun L. (2017). IRF9 inhibits human acute myeloid leukemia through the SIRT1-p53 signaling pathway. FEBS Lett..

[52] Park S.H., Kang K., Giannopoulou E., Qiao Y., Kang K., Kim G., Park-Min K.H., Ivashkiv L.B. (2017). Type I interferons and the cytokine TNF cooperatively reprogram the macrophage epigenome to promote inflammatory activation. Nat. Immunol..

