# Supplementary material for: The Combination of IFN β and TNF Induces an Antiviral and Immunoregulatory Program via Non-Canonical Pathways Involving STAT2 and IRF9
Source: Cells. 2019 Aug 17;8(8):919. doi: 10.3390/cells8080919 (PMC6721756; doi:10.3390/cells8080919)
Supplement: Supplementary file 1 [file cells-08-00919-s001.zip › Supplemental Figures revised/Supplemental-Figure S1-revised.pdf]

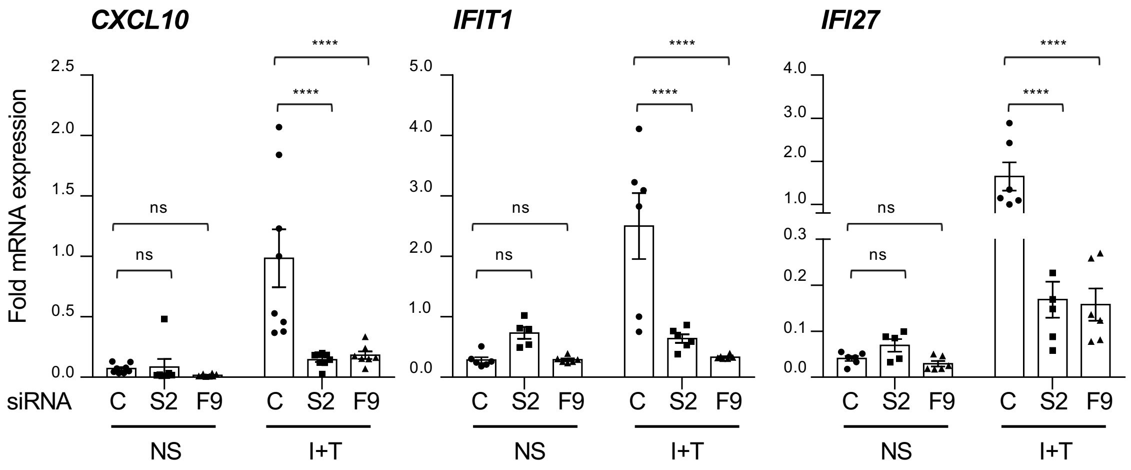

Supplemental Figure S1. qRT-PCR analysis of IFNβ+TNF (I+T) upregulated DEGs positively regulated by STAT2 and IRF9. U3A cells were transfected with siCTRL (C), siSTAT2 (S2) or siIRF9 (F9). Cells were further left untreated or stimulated with I+T for 24h. Quantification of the mRNA corresponding to the indicated genes was performed by qRT-PCR and expressed as relative expression (DDCt) after normalization to the S9 mRNA levels. Mean +/- SEM, n≥5. Statistical comparison was conducted using Two-way ANOVA with tukey post-test. $p < 0.0001$ (****); ns: not significant.
