# Supplementary material for: The Combination of IFN β and TNF Induces an Antiviral and Immunoregulatory Program via Non-Canonical Pathways Involving STAT2 and IRF9
Source: Cells. 2019 Aug 17;8(8):919. doi: 10.3390/cells8080919 (PMC6721756; doi:10.3390/cells8080919)
Supplement: Supplementary file 1 [file cells-08-00919-s001.zip › Supplemental Figures revised/Supplemental Table S4-MatetMeth-qPCR-revised.docx]

| **SYBR Green** | | |
| --- | --- | --- |
| **Gene** | **Sense (S) /Antisense (AS)** | **Sequence (5’-3’)** |
| *APOBEC3G* | S | GGTCAGAGGACGGCATGAGA |
|  | AS | GCAGGACCCAGGTGTCATTG |
| *IDO* | S | taccatctgcaaatcgtgactaagt |
|  | AS | gaagggtcttcagaggtcttattctc |
| *CXCL10* | S | gaaattattcctgcaagccaattt |
|  | AS | tcacccttctttttcattgtagca |
| *NOD2* | S | agccattgtcaggaggctc |
|  | AS | cgtctctgctccatcatagg |
| *IRF1* | S | ttccctcttccactcggagt |
|  | AS | gatatctggcagggagttca |
| *PKR* | S | tcttcatgtatgtgacactgc |
|  | AS | cacacagtcaaggtccttag |
| *MX1* | S | attcggatgcttcagaggtaga |
|  | AS | cccggcgatggcatt |
| *S9* | S | attcggatgcttcagaggtaga |
|  | AS | cccggcgatggcatt |
| *IL8* | S | TCTCTTGGCAGCCTTCCTGATTTC |
|  | AS | GTGTGGTCCACTCTCAATCACTCT |
| *IFIT1* | S | GCCCAGACTTACCTGGACAA |
|  | AS | GGTTTTCAGGGTCCACTTCA |
| **Taqman probes** | | |
| **Gene** |  | **Applied Biosystems Assays Catalog number** |
| *CCL20* |  | Hs00355476_m1 |
| *DUOX2* |  | Hs00204187_m1 |
| *IL33* |  | Hs01125944_g1 |
| *ISG20* |  | Hs00158122_m1 |
| *IFI27* |  | Hs01086373_g1 |

**Supplemental Table S4** Primer sequences and Taqman probes used in qRT-PCR analysis
